# Metabolites as drivers and targets in rheumatoid arthritis

**DOI:** 10.1093/cei/uxab021

**Published:** 2021-11-27

**Authors:** Megan M Hanlon, Mary Canavan, Brianne E Barker, Ursula Fearon

**Affiliations:** Molecular Rheumatology, Trinity Biomedical Sciences Institute, Trinity College Dublin, Dublin, Ireland; EULAR Centre of Excellence for Rheumatology, Centre for Arthritis and Rheumatic Diseases, St. Vincent’s University Hospital, Dublin, Ireland; Molecular Rheumatology, Trinity Biomedical Sciences Institute, Trinity College Dublin, Dublin, Ireland; EULAR Centre of Excellence for Rheumatology, Centre for Arthritis and Rheumatic Diseases, St. Vincent’s University Hospital, Dublin, Ireland; Molecular Rheumatology, Trinity Biomedical Sciences Institute, Trinity College Dublin, Dublin, Ireland; EULAR Centre of Excellence for Rheumatology, Centre for Arthritis and Rheumatic Diseases, St. Vincent’s University Hospital, Dublin, Ireland; Molecular Rheumatology, Trinity Biomedical Sciences Institute, Trinity College Dublin, Dublin, Ireland; EULAR Centre of Excellence for Rheumatology, Centre for Arthritis and Rheumatic Diseases, St. Vincent’s University Hospital, Dublin, Ireland

**Keywords:** rheumatoid arthritis, synovial tissue, metabolites, glycolysis, oxidative phosphorylation

## Abstract

Rheumatoid arthritis (RA) is a chronic autoimmune disease characterized by neovascularization, immune cell infiltration, and synovial hyperplasia, which leads to degradation of articular cartilage and bone, and subsequent functional disability. Dysregulated angiogenesis, synovial hypoxia, and immune cell infiltration result in a ‘bioenergetic crisis’ in the inflamed joint which further exacerbates synovial invasiveness. Several studies have examined this vicious cycle between metabolism, immunity, and inflammation and the role metabolites play in these interactions. To add to this complexity, the inflamed synovium is a multicellular tissue with many cellular subsets having different metabolic requirements. Metabolites can shape the inflammatory phenotype of immune cell subsets during disease and act as central signalling hubs. In the RA joint, the increased energy demand of stromal and immune cells leads to the accumulation of metabolites such as lactate, citrate, and succinate as well as adipocytokines which can regulate downstream signalling pathways. Transcription factors such as HIF1ɑ and mTOR can act as metabolic sensors to activate synovial cells and drive pro-inflammatory effector function, thus perpetuating chronic inflammation further. These metabolic intermediates may be potential therapeutic targets and so understanding the complex interplay between metabolites and synovial cells in RA may allow for identification of novel therapeutic strategies but also may provide significant insight into the underlying mechanisms of disease pathogenesis.

## Teaser

The adverse microenvironment of the RA inflamed joint forces cells to switch their metabolic pathways in order to sustain their pathogenic activated status. Metabolites act as central hubs influencing multiple cellular functions and signaling pathways that further drive inflammation.

## Introduction

Rheumatoid arthritis (RA) is a chronic progressive autoimmune disease, characterized by neo-angiogenesis, immune-cell infiltrates, secretion of pro-inflammatory mediators, and activation of synovial fibroblasts, leading to subsequent degradation of articular cartilage and bone, and thus functional disability [1]. The blood vessels of the healthy synovium are lined with a monolayer of quiescent endothelial cells organized as a ‘phalanx’ aligned in the direction of blood flow which supply nutrients and oxygen to the synovium. However, during inflammation, activated endothelial cells lose their polarity, detach and protrude into the vessel lumen, leading to abnormal synovial vascular morphology [2–8]. Numerous studies have demonstrated distinct macroscopic vascular patterns in the inflamed joint, which is paralleled at the microscopic level by an increase in the number of blood vessels, in addition to differential expression of key vascular growth factors^-^associated with sprout formation [2–8]. The poorly organized vasculature limits the efficient delivery of nutrients and oxygen to the joint. This along with the increased metabolic demand of activated immune and stromal cells within the synovial compartment leads to a hypoxic joint microenvironment [3]. This forces synovial cells to adapt to this adverse microenvironment by switching their metabolic pathways in order to sustain their pathogenic activated status, with synovial cell types displaying differing nutrient demands. This creates a vicious ‘bioenergetic crisis’ within the inflamed joint which further potentiates synovial invasiveness within the RA joint [3, 8–10].

**Figure 1:**
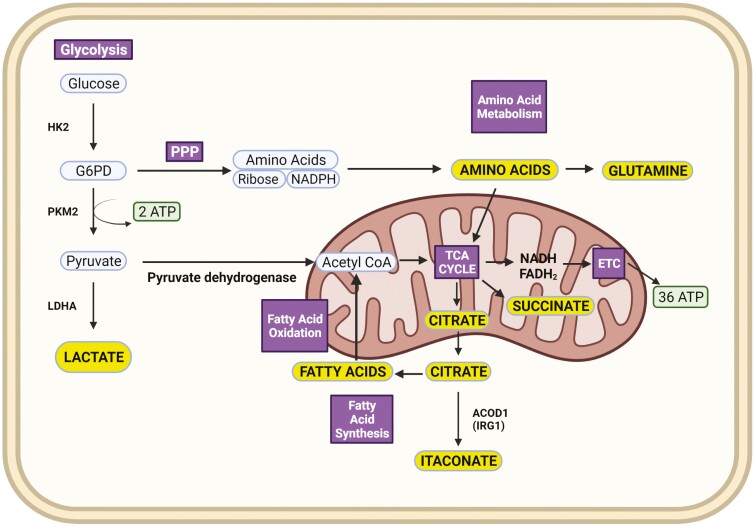
Overview of the main metabolic pathways: Glucose enters the cell via glucose transporters and enters the glycolytic pathway. Hexokinase 2 (HK2) converts glucose into Glucose 6-phosphate dehydrogenase (G6PD). Glycolysis generates pyruvate from glucose with the help of Pyruvate Kinase M2 (PKM2). This process generates energy in the form of adenosine tri-phosphate (ATP). Pyruvate is then either converted to lactate and secreted out of the cell or decarboxylated by pyruvate dehydrogenase (PDH) and converted to Acetyl CoA which enters the Tricarboxylic Acid (TCA) cycle. The TCA cycle generates NADH, FADH2 to feed into the Electron Transport Chain (ETC), which produces 36 molecules of ATP. Glycolysis also feeds into the Pentose Phosphate Pathway (PPP) at the level of G6PD to produce ribose, NADPH, and amino acids. Amino acid metabolism can also feed into the TCA cycle to drive ATP production by the ETC, amino acids such as Glutamine can have further downstream effects. TCA intermediate citrate drives fatty acid synthesis while fatty acid oxidation drives TCA cycle further by generating Acetyl CoA. Citrate and Succinate can act as ‘immunometabolites’. Citrate can also be converted to Itaconate via the enzymatic activity of IRG1 (ACOD1). This figure was created using biorender.com.

While an emerging body of evidence over the last 10 years has highlighted the role of altered cellular metabolism in the pathogenesis of RA (Fig. 1), the concept was originally hypothesized in the 1970s where studies demonstrated increases in the level of metabolites in synovial fluid and in the activity of glyceraldehyde 3-phosphate dehydrogenase and lactate dehydrogenase in RA synovial cells [11, 12]. Subsequent studies using proton magnetic resonance spectroscopy (MRS) demonstrated marked increases in lactate levels paralleled by a decrease in glucose concentration in RA synovial fluids [13, 14], with increased lactic acid levels in the joint shown to correlate with cytokines and disease activity scores, indicating a direct link between altered metabolism and level of inflammation [13, 14]. These original findings are supported by recent studies using metabolomics [15–17], in addition to studies which performed direct measurement of synovial tissue pO2 levels using a Lycox Probe, which demonstrated that the RA joint is profoundly hypoxic [18]. Joint hypoxia was associated with increased lactate levels [10], and inversely correlated with synovial inflammation and treatment response [18, 19]. Consistent with this, increased expression of the master regulator of hypoxia ‘hypoxia-inducible factor (HIF)-1alpha’ is expressed in both the lining and sub-lining cells of the synovium [20, 21], with numerous studies showing that HIF1α activation can regulate expression of key transcriptional factors and metabolic enzymes, which in turn promote synovial pathogenic mechanisms [21–27].

### Lactate

**Figure 2:**
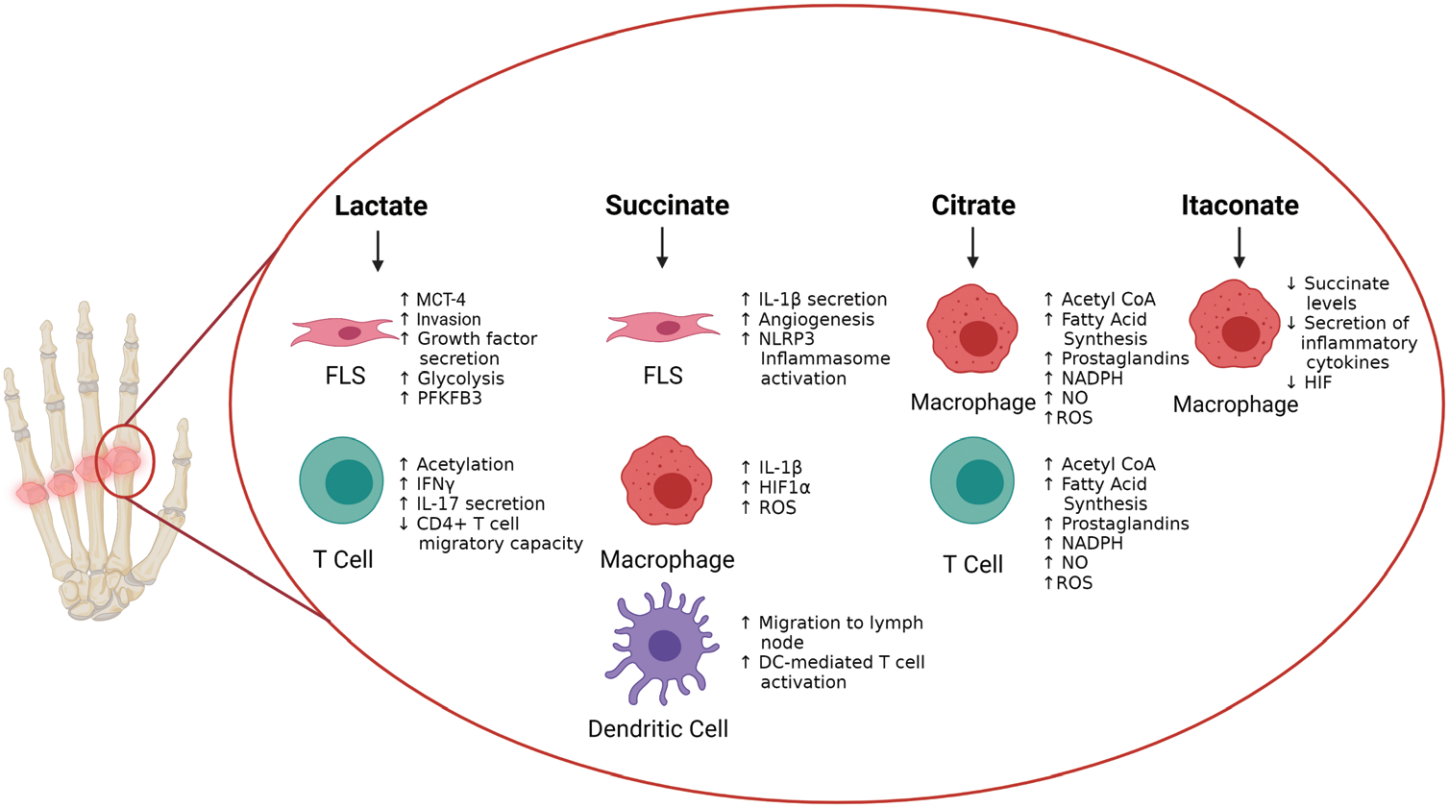
Effect of ‘immunometabolites’ on specific cell subsets within the inflamed synovial joint. During the inflammatory process, immune and stromal cells at the site of inflammation sense and respond to elevated levels of metabolites such as lactate, citrate, succinate, and itaconate. RA FLS express high levels of lactate transporter MCT-4 to facilitate export of lactate into extracellular space. Lactate induces increased invasiveness, growth factor secretion, enhanced glycolysis and glycolytic enzymes in RA FLS. Lactate also acts on synovial T cells to induce increased acetylation, IFNϒ and IL-17 secretion with a decrease in CD4+ T-cell migratory capacity to ‘entrap’ T cells at the site of inflammation. Succinate acting on RA FLS results in IL-1B secretion, activation of the inflammasome, and increased angiogenesis while macrophages respond to succinate through increased HIF1α, ROS and IL-1B production. Activation of the succinate receptor on dendritic cells orchestrates their migration to the lymph node and enhances DC-mediated antigen-specific T-cell activation. Citrate induces increased expression of NADPH, ROS, NO, prostaglandins, and fatty acid synthesis in both macrophages and T cells. Itaconate has immunomodulatory effects on activated macrophages resulting in decreased secretion of pro-inflammatory cytokines, HIF1α production and levels of succinate. This figure was created using biorender.com.

Lactate is an important metabolite produced at the end of the glycolytic process under hypoxic conditions. While several studies have demonstrated the accumulation of lactate in the inflamed joint [10, 12–17, 28, 29], it is evident that it is not simply a by-product of metabolism but can also further potentiate the inflammatory response (Fig. 2). Recent studies have shown that quiescent RA synovial fibroblasts (FLS) produce significantly higher levels of lactate compared to osteoarthritis FLS [30]. In addition, condition media from T cells induce a glycolytic shift in RA FLS towards aerobic glycolysis, with significant increases in lactate production observed [30]. Active endothelial cells which line synovial blood vessels also rely heavily on glycolysis [31, 32], further contributing to lactate accumulation in the RA joint. At a functional level, studies have shown that RA FLS express high levels of the lactate transporter MCT-4 facilitating export of intracellular lactate into the extracellular space [29], that culture of RA FLS with lactate induces RA FLS invasion and secretion of growth factors [10], and that MCT-4 knockdown promotes RA FLS apoptosis and reduction in the severity of arthritis in a collagen-induced arthritis (CIA) model [29]. Furthermore, studies have demonstrated that blockade of the glycolytic enzyme PFKFB3 which leads to a decrease in RA FLS proliferation, migration, and invasive capacity is reversed in the presence of lactate, an effect mediated via PFKFB3-induced transcriptional activation of NF-κB and MAPKs [33].

The key role for lactate dehydrogenase A (LDHA) in the regulation of inflammation has also been demonstrated in T cells, where a study by Peng *et al.* demonstrated LDHA mediates the induction of T-cell effector function through increased acetylation and IFN-γ transcript [34]. Studies have shown that RA synovial tissue expression of the lactate transporter SLC5a12 correlates with the clinical T-cell score and with the formation of ectopic lymphoid structures [35]. This study also showed that sodium lactate regulates T-cell effector function resulting in increased IL-17 secretion and reduced migratory capacity of CD4+ T cells [35]. In contrast, this study also showed that lactic acid led to a loss of cytolytic function in RA CD8+ T cells [35]. This effect was mediated via inhibition of the glycolytic enzymes HK and PFK, with *in-vivo* antibody blockade of lactate transporters leading to the release of T cells from the inflammatory site [35]. More recently, the same group went on to further demonstrate that the modulatory effect of the lactate transporter SLC5A12 on CD4^+^ T-cell entrapment was mediated via nuclear PKM2/STAT3 interactions, reduced glycolysis, and enhanced fatty acid synthesis (FAS). Furthermore, SLC5A12 antibody blockade led to amelioration in the severity of arthritis in a murine model of arthritis [36]. These studies provide a rationale for the therapeutic targeting of specific lactate transporters on T cells in RA; indeed, LDHA inhibition with GSK2837808A has been shown to restore effector T-cell functions even in the presence of lactate [37].

### TCA cycle metabolites

The tricarboxylic acid (TCA) cycle is an important metabolic pathway that takes place in the mitochondrial matrix whereby a series of enzymatic reactions occur resulting in generation of NADH and FADH2 which then fuels the electron transport chain. Metabolomic analysis of synovial fluid from RA patients has demonstrated elevated levels of many intermediates of the TCA cycle, particularly succinate and citrate compared with other inflammatory arthritides [28], which suggests these molecules may be ‘immunometabolites’ capable of shaping effector functions of immune cells in RA (Fig. 2).

Succinate, a small dicarboxylic acid, is a key metabolite intersecting many metabolic pathways. Succinate is produced by succinyl CoA and metabolized to fumarate by succinate dehydrogenase. Abundant amounts of extracellular succinate have been detected in RA synovial fluid with a global metabolic profiling study identifying it as the most differentially expressed metabolite in RA compared with other forms of inflammatory arthritis [28]. In inflammatory conditions such as RA, activated macrophages accumulate succinate following a distinct break in the TCA cycle. Accumulation of succinate causes stabilization of HIF1α which subsequently promotes IL-1β production [38]. Moreover, succinate activates the NLRP3 inflammasome and induces IL-1β secretion in synovial fibroblasts in a collagen-induced arthritis rat model [39]. Succinate has also been shown to induce synovial angiogenesis through VEGF-dependent HIF1α pathways in RA mouse models [40]. An increase in synovial angiogenesis not only supplies more oxygen and nutrients to the inflamed joint but also promotes pannus formation by facilitating inflammatory cell migration [40].

Succinate is the endogenous ligand for the receptor GPR91; indeed studies have shown that activation of GPR91 in macrophages results in release of succinate into the extracellular milieu in antigen-induced arthritis [41]. Moreover, succinate perpetuates the inflammatory response through HIF1α-induced IL-1β production and generation of reactive oxygen species (ROS) in inflammatory macrophages [38, 42], in a GPR91-dependant manner [41]. Not surprisingly, mice lacking GPR91 show reduced macrophage activation and IL-1β secretion in a model of antigen-induced arthritis [41]. Activation of the succinate receptor on dendritic cells (DCs) orchestrates their migration to the lymph node and enhances DC-mediated antigen-specific T-cell activation. This GPR91-mediated chemotactic DC signal is specifically linked to Th1 expansion and exacerbation of antigen-induced arthritis [43].

The Krebs cycle begins with the aldol condensation of oxaloacetate and acetyl CoA via citrate synthase to form citrate [44]. Much like succinate, citrate also accumulates in inflammatory macrophages due to a TCA cycle break with levels elevated in synovial fluid of RA patients compared with other forms of inflammatory arthritis [28]. Accumulation of citrate is also observed in activated CD4+ RA T cells due to increased lactate uptake and metabolism [36]. Citrate can be exported from the mitochondria, where it may be converted back to acetyl-CoA and then serve as a substrate for fatty acid synthesis and lipids such as prostaglandins. Citrate export from the mitochondria can provide a source of NADPH which is required for the generation of NO and ROS [45].

Directly, citrate can promote pro-inflammatory mechanisms but also indirectly modulates inflammation through the effects of itaconate. Citrate can be converted to itaconate by the enzyme aconitate decarboxylase 1 (ACOD1) also known as IRG1. IRG1 is one of the most robustly induced genes following LPS stimulation of macrophages [46]. Itaconate has also been shown to be elevated in the RA joint and positively correlates to disease activity and anti-hTNF biologic infliximab treatment response in arthritis models [47]. Moreover, itaconate levels have been detected in the plasma of early RA patients with a decrease in overall disease activity associated with increased plasma itaconate levels [48]. Originally, the key function of itaconate’s immunomodulatory effect was thought to be its ability to inhibit succinate oxidation by succinate dehydrogenase [49]. Recently, however, it has been shown that itaconate can indirectly activate the anti-inflammatory transcription factor Nrf2 via alkylation of KEAP1 in the cytosol [50, 51]. Itaconate has been identified as a key marker of disease activity in a mouse model of arthritis and interestingly elevated itaconate levels can be reversed upon TNF blockade with infliximab [47]. This is in contrast with studies in RA patients whereby increased itaconate is associated with changes in disease activity following the initial 3 months of conventional DMARD therapy [48]. These conflicting studies suggest that itaconate complex responses are tissue and context dependent. Itaconate also regulates succinate levels, secretion of inflammatory cytokines, and HIF1α in inflammatory macrophages [49]. Therefore, itaconate is seen as a key anti-inflammatory metabolite, induced by inflammatory stimuli, in the context of rheumatoid arthritis.

### Lipid metabolites

**Figure 3:**
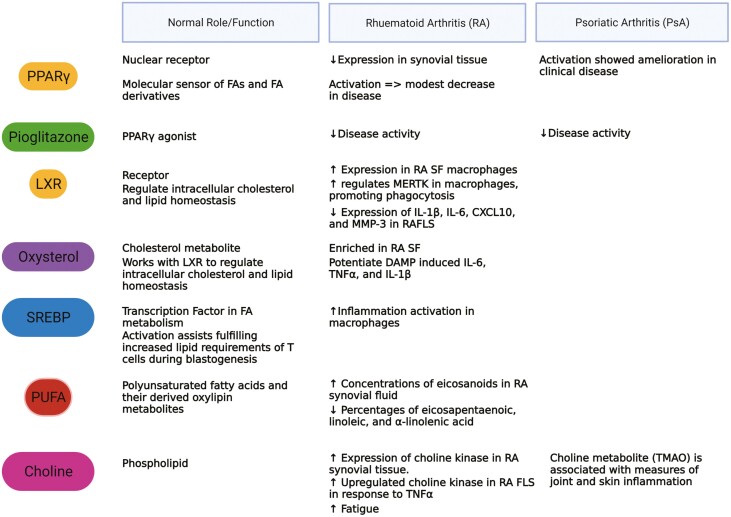
Overview of lipid metabolites and their functions in healthy, rheumatoid and psoriatic arthritis. Role of lipid metabolites such as oxysterol, choline, and PUFA and key receptors PPARϒ, SREBP, and LXR in normal physiology and the role they play in RA and PsA pathogenesis. The nuclear receptor PPARϒ is decreased in RA synovial tissue and upon activation improves PsA disease outcomes. Its agonist Pioglitazone also decreases disease activity both in RA and PsA. LXR, a receptor that regulates intracellular cholesterol and lipid homeostasis, is increased in RA synovial fluid macrophages yet can conversely decrease the inflammatory and invasive capacity of RA FLS. Oxysterold works with LXR to regulate lipid homeostasis and is also enriched in RA synovial fluid. The transcription factor SREBP induces activation of inflammatory macrophages while polyunsaturated fatty acids are increased in RA compared to OA. Choline and its metabolite TMAO is associated with measures of joint and skin inflammation in PsA and there is enriched expression of choline kinase in RA synovial tissue and RA FLS in response to TNFα.

Lipid metabolism is an essential biochemical process involving the production and the breakdown of lipid species such as fatty acids (FAs), cholesterol, and phospholipids. These lipids carry out a range of physiological processes in the body such as membrane biosynthesis, cell signalling, energy storage, and hormone development [52]. However, in recent years, it has now emerged that lipids and lipid metabolism may also have an essential and non-redundant role in inflammation and immunity [53, 54]. Moreover, metabolites of these pathways have been implicated in autoimmunity, where both pro- and anti-inflammatory effects have been described (Fig. 3).

Transcriptional regulation of lipid metabolism is tightly controlled by receptors such as peroxisome proliferator-activated receptors (PPAR) and liver X receptors (LXR) and the sterol regulatory element binding proteins (SREBP). The cholesterol metabolite oxysterol and its receptor LXR are important regulators of intracellular cholesterol and lipid homeostasis and have previously been implicated in IA pathogenesis. Previous studies have demonstrated that the LXR pathway is highly upregulated in RA synovial fluid (SF) macrophages, while oxysterols, which are enriched in RA synovial fluid, potentiate DAMP-induced TNF, IL-6, and IL-1β production [55]. Moreover, LXR promotes phagocytosis by upregulating MERTK expression in macrophages [56], while previous studies have also demonstrated an increase in MERTK-positive macrophages in RA synovial tissue. However, conflicting data exist on the role of LXR in other cell types, specifically synovial fibroblasts. LXR agonists decreased the expression of IL-1β, IL-6, CXCL10, and MMP-3 from RA synovial fibroblasts in addition to abrogating their invasive capacity [57]. Cholesterol itself has also been shown to be a risk factor for the development of RA in women but not men, suggesting that hormone-specific factors may also modulate the impact of lipids in RA [58]. In addition to cholesterol and cholesterol metabolites, other lipid metabolites such as FA have also proven to display both pro- or anti-inflammatory phenotypes, effects which appear to be reliant on the cell type involved or the unique properties of individual metabolites.

FA metabolism is controlled by nuclear receptors known as PPARs. PPARs consist of three subtypes PPARα, PPARδ, and PPARƴ, all of which act as molecular sensors of FAs and FA derivatives [59]. PPARγ expression is significantly decreased within RA synovial tissue compared with both osteoarthritis (OA) and normal tissue [60]. Moreover, the PPARγ agonist, pioglitazone has previously been used in pilot studies in the treatment of both psoriatic arthritis (PsA) [61] and RA [62]. Activation of PPARƴ modestly reduced RA disease activity as measured by DAS28-CRP, while amelioration in clinical disease was also reported in PsA. While direct evidence for the role of SREBP1 and SREBP2 has yet to be established in IA, multiple studies have highlighted an immunological role for this transcription factor. Specifically, clonally expanded T cells have increased lipid requirements during blastogenesis, a requirement which extensively relies on SREBP activation [63]. Furthermore, SREBP2 can drive inflammasome activation in macrophages, thus supporting the development of an M1-like proinflammatory macrophage subtype [64]. While studies examining the specific contribution of SREBP in IA have yet to be explored, a single-nucleotide polymorphism in SREBP-2 was demonstrated to be significantly associated with the development of OA [65]. Collectively, these early data suggest that activation of PPAR receptors may prove beneficial in ameliorating inflammation, while alternatively SREBP, another major transcription factor in FA metabolism may contribute to inflammatory processes in the joint.

Interestingly, oxylipin metabolites derived from polyunsaturated fatty acid (PUFA) have also been examined in the context of RA. PUFA-derived oxylipins include classic eicosanoids (prostaglandins, thromboxanes, and leukotrienes) in addition to pro-resolving lipid mediators (lipoxins, resolvins, and protectins) [66]. A recent lipidomic study by Sano *et al*. demonstrated that concentrations of these pro-inflammatory eicosanoids in addition to specialized pro-resolving lipid mediators were both significantly higher in RA synovial fluid compared to OA [67]. Moreover, among PUFA themselves, decreased percentages of linoleic acid, α-linolenic acid, and eicosapentaenoic acid have been reported in RA compared to OA SF [68].

The phospholipid choline and its metabolite trimethylamine-N-oxide (TMAO) have also been implicated in IA disease progression. The choline kinase ChoKα is expressed in RA synovial tissue and is upregulated in RA FLS in response to TNFα. Furthermore, inhibition of ChoKα in RA FLS decreased cell migration and resistance to apoptosis [69]. Serum TMAO is also associated with measures of joint (tender joint count, swollen joint count, DAS28-CRP) and skin inflammation (body surface area affected by psoriasis) in patients suffering from PsA or psoriasis [70]. Interestingly, upon examination of choline using whole brain MRS imaging in RA patients, fatigue severity was positively correlated with the presence of choline in several brain regions suggesting that alterations in lipid metabolism may have multiple disease-related effects in IA [71]. Collectively, these data suggest that lipid metabolites may contribute to both the activation of inflammation and indeed anti-inflammatory resolution pathways, further emphasizing the need to further work in this area.

### Amino acids

The crosstalk between catabolism of amino acids and the immune response has emerged as a vital process that controls the regulation of inflammation. Metabolic profiling of plasma revealed significantly altered amino acid and nicotinamide metabolites in RA when compared with healthy control and PsA plasma [72]. Indeed, altered amino acid metabolism was one of the initial discriminatory markers to define polarized macrophages. LPS/IFNy pro-inflammatory macrophages (M1-like) convert arginine to nitric oxide (NO) via inducible NO synthase (iNOS) while alternatively activated IL-4 macrophages (M2-like) metabolizes arginine by arginase-1 [73, 74]. The imbalance between M1-like and M2-like macrophages, favouring the pro-inflammatory M1-like cells, is a major driver of RA disease progression. Metabolism of arginine to release nitric oxide by iNOS can have pleiotropic effects depending on cell type. NO production in inflammatory macrophages and DCs acts to inhibit mitochondrial respiration to drive a pro-glycolytic phenotype [75, 76]. However, arginine metabolism in T cells instead promotes a metabolic switch to OXPHOS and exerts anti-inflammatory effects [77, 78].

Glutamine is a key amino acid involved in the TCA cycle and the main nitrogen donor for the production of nucleic acids and non-essential amino acids. Glutamine has been shown to be highly elevated in synovial fluid of RA patients compared with other inflammatory conditions [28]. Glutamine provides carbons to the TCA cycle in a process called glutaminolysis whereby glutamine is first converted to glutamate and then a-ketoglutarate [79]. Glutaminase 1 (GLS1) is the enzyme involved in catalysing the first step of this process and has been shown to be elevated in RA FLS, inhibition of which significantly reduces RA FLS proliferation and ameliorates disease in a murine model of arthritis [80]. These results suggest that RA FLS may be ‘addicted to glutamine’ reminiscent of what has been described in certain cancer cells [81]. Moreover, glutamine has been shown to regulate the balance between Tregs and Th1/Th17 cells with evidence showing that GLS1 is upregulated in Th17 cells and they are more reliant on glutaminolysis compared with other T-cell subsets [82–84]. Using the glutamine antagonist 6-diazo-5-oxo-L-norleucine reduced Th17 splenic cells and suppressed arthritis when in combination with rapamycin (mTOR inhibitor) in a mouse model of disease [85].

Amino acid transporters have also been implicated in inflammation. SLC7A5 is a crucial transporter that mediates uptake of many essential amino acids. Studies have shown that SLC7A5 induces proinflammatory cytokine secretion in RA monocytes and macrophages through leucine influx and has also been demonstrated to play a key role in tumour survival and growth [86–88]. Indeed elevated expression of SLC7A5 in RA monocytes correlates with both C-reactive protein (CRP) and erythrocyte sedimentation rate, thus suggesting that SLC7A5-mediated amino acid influx is associated with RA disease pathogenesis [86].

Tryptophan is an essential amino acid that is a precursor for the synthesis of proteins and many molecules including kynurenine, NAD, serotonin, and melatonin [89]. IDO mediates the initial rate-limiting step in the catabolism of tryptophan to kynurenine [90]. Studies have demonstrated that IDO2 but not IDO1 is required for activation of CD4+ T cells, autoantibody production, and development of disease in a mouse model of arthritis [91]. Moreover, treatment with anti-IDO2 antibodies inhibits autoreactive T- and B-cell responses and alleviates joint inflammation and severity of RA disease [91–93]. In fact studies into animal models of arthritis have shown that combining an IDO inhibitor with either methotrexate (MTX) or B-cell depletion therapy was more effective in alleviating arthritis compared with either treatment alone [94, 95]. In contrast, studies have also demonstrated that IDO inhibition accelerates CIA and enhances immune responses in a mouse model. Targeting IDO-mediated tryptophan catabolism in this study resulted in Th1 cell accumulation [96]. Indeed, intra-articular delivery of IDO into the ankles of CIA rats resulted in amelioration of arthritis and reduction of synovial IL-17 production suggesting a role for IDO gene therapy [97]. TLR-4-mediated IDO expression has been shown to be elevated following depletion of MyD88 in RA synovial fibroblasts [98] while RA or OA synovial fibroblasts are capable of suppressing Th cell responses via IDO1-mediated tryptophan deletion [99]. Moreover, tryptophan can exert anti-inflammatory effects through its own metabolites such as kynurenine. Kynurenine itself is a ligand for the aryl hydrocarbon receptor, implicated in immune cell maturation and promotes Treg differentiation while simultaneously suppressing Th17 differentiation [100].

Tryptophan is also a precursor for NAD+, an important hydrogen carrier in mitochondrial respiration. NAD+ can be synthesized either through the *de novo* synthesis pathway or via the salvage pathway. Nicotinamide phosphoribosyltransferase (NAMPT) is a rate-limiting enzyme in the NAD+ salvage pathway and is increased in the RA serum and fluid, positively correlating with disease activity and radiographic progression [101–103]. Also called visfatin, extracellular NAMPT has been defined as a metabokine as well as an adipokine given its release from adipocytes [104, 105]. NAMPT correlates with induced expression of pro-inflammatory mediators in monocytes and studies have reported that NAMPT inhibition decreases the infiltration and activation of immune cells into arthritic joints in a CIA model [101, 106]. Inhibition of NAMPT using the small molecule inhibitor FK866 (also known as APO866) has been shown to reduce synovial inflammation and bone erosion as well as many systemic markers of inflammation in mouse models of arthritis [107, 108].

A recent study demonstrated decreased levels of many amino acids including tryptophan and glycine in RA patients compared to healthy controls; treatment with MTX returned amino acid levels to baseline [109]. Similarly, another study indicated increases in serum amino acids such as leucine, valine, alanine, and glutamine in RA patients treated with a TNF inhibitor; elevated amino acid plasma levels suggest decreased amino acid metabolism in RA responders [110, 111]. This is consistent with studies demonstrating decreased amino acid levels in RA synovial fibroblasts compared to OA, perhaps an indication of amino acids being used as energy substrates for gluconeogenesis in RA [112].

## Metabolic sensors

### Hypoxia-inducible factor

**Figure 4:**
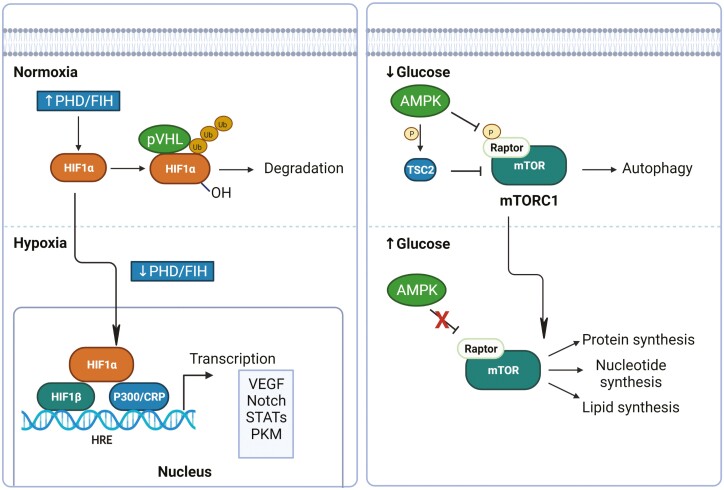
HIF1α and AMPK signalling in the rheumatoid joint. Under normoxic conditions, hydroxylation of hypoxia-inducible factor (HIF)-1α by prolyl hydroxylases (PHDs) generates a binding site for the von Hippel Lindau tumour suppressor protein (VHL), thereby promoting the ubiquitylation and subsequent proteasomal degradation of HIF-1α. However, under hypoxic conditions, such as in the inflamed joint, the hydroxylation activity of PHDs is reduced, resulting in the accumulation and activation of HIF-1α, which can then translocate into the nucleus and associate with HIF-1β and the cofactor p300/CBP. This complex binds to, and induces the transcription of genes such as VEGF, Notch, STAT3, and PKM2. In glucose deprived environments, the energy sensor AMPK inhibits mTORC1 through the phosphorylation and activation of the mTOR negative regulator tuberous sclerosis complex 2 (TSC2), and through the phosphorylation and inhibition of the mTORC1 component regulatory-associated protein of mTOR (Raptor). However, in environments of high glucose availability, this process does not occur allowing mTORC1 to be phosphorylated and activates a number of downstream targets including protein, nucleotide, and lipid synthesis, while blocking the catabolic processes such as autophagy. This figure was created using biorender.com.

The RA joint microenvironment is hypoxic, as highlighted above, with synovial pO2 levels as low as 0.46% in some RA patients [18]. Activation of the master regulator ‘HIF’ executes our cellular response to altered oxygen [113]. HIF is composed of two subunits, HIF1α, which is regulated indirectly by oxygen availability, and HIF1β which is constitutively expressed in the nucleus of the cell [114]. Under hypoxic conditions, HIF1α subunits rapidly increase, an effect that is regulated via the oxygen-dependent hydroxylase enzymes [114] (Fig. 4). These include three prolyl hydroxylases (PHD1, PHD2, and PHD3) and one asparagine hydroxylase factor inhibiting HIF (FIH), which belong to the 2-oxoglutarate-dependent iron II dioxygenase super family [114]. Under normoxic conditions, PHDs hydroxylate two prolyl residues on HIF1α (pro402 and pro564), making available the binding site for von Hippel Lindau tumor suppressor protein (pVHL) [115], which in turn results in polyubiquitylation of HIF1α and subsequent proteasomal degradation. However, under hypoxic conditions, the activity of the hydroxylase enzymes is inhibited, leading to accumulation of HIF1α and subsequent translocation to the nucleus where it dimerizes with HIF1β and its cofactor p300/CBP (Fig. 4). This HIF1α complex binds to specific DNA motifs (hypoxia-responsive elements) and induces transcriptional activation of hundreds of genes involved in survival, immune effector cell function, metabolism, angiogenesis, and invasion (Fig. 4).

Numerous studies have shown increased HIF1α and 2α expression in the RA synovium, localized to both the lining layer and sub-lining regions, with level of expression associated with increased inflammation [20, 116]. Furthermore, HIF1α expression has been observed in the synovium at a pre-clinical stage of disease in animal models [117] and in monocytes from individuals ‘at-risk’ of developing RA [118]. At a functional level, HIF1α overexpression enhances RA FLS-mediated expansion of inflammatory Th1 and Th17 cells [118, 119]. Hypoxia potentiates the effects of IL-17A, IL-1β, and TNFα on angiogenic and invasive mechanisms in RA FLS via interaction of HIF1α and NF-κB signalling [119, 120]. In turn, IL-17 and TNFα synergistically induce a HIF1α-dependent invasive phenotype in RA FLS [121], with HIF1α signalling also shown to mediate TLR-induced RA FLS function [119]. More recently, direct interaction between HIF1α signalling and metabolites has also been shown, where succinate accumulation in the inflamed joint activates the NLRP3 inflammasome and subsequent IL-1β secretion, via HIF1α signalling [39]. Furthermore, intracellular succinate acts through HIF1α signalling, while extracellular succinate acts via the GPR91 receptor, which together alter metabolic, angiogenic, and inflammatory responses in the inflamed joint synovium [40]. HIF1α is also an upstream regulator of the glycolytic enzymes glucose-6-phosphate isomerase and glyceraldehyde-3-phosphate dehydrogenase, both of which induce invasive function in RA FLS [122]. Furthermore, FcγR-mediated metabolic reprogramming in macrophages is mediated via HIF1α and mTOR activation [123], with studies also demonstrating that LPS-induced PKM2 in macrophages forms a complex with HIF1α which in turn promotes IL-1β secretion [124]. Finally, numerous studies in animal models have demonstrated that loss of HIF1α results in amelioration of inflammation [125, 126].

*In vivo* synovial pO2 levels are also associated with increased activation of JAK-STAT, a key signalling pathway implicated in the pathogenesis of RA. STAT3 activation has been shown to mediate hypoxia-induced RA FLS migration and invasive mechanisms, effects of which are inhibited by HIF1α silencing [25]. In turn, STAT inhibition or siRNA can also inhibit hypoxia-induced HIF1α expression, suggesting bi-directional interactions [25]. This is consistent with studies showing hypoxia-induced STAT3 activation can accelerate HIF1α protein accumulation/activation [127], an effect mediated through competition of STAT3 with HIF1α for pVHL binding [128]. The JAK inhibitor WP1066 significantly decreases hypoxia-induced HIF1α and Notch-1IC signalling in RA FLS, resulting in reduction in RA FLS invasive capacity [25]. Interactions between STAT3 and metabolic enzymes have also been demonstrated previously whereby blockade of PFKFB3 leads to inhibition of pSTAT3 expression in RA FLS [10], and nuclear PKM2 can act as a protein kinase which phosphorylates STAT3 transcription which in turn promotes IL-6 and IL-1β secretion [129]. SLC5A12-mediated lactate uptake in CD4+ T cells leads to increased IL17 production via nuclear PKM2/STAT3 and enhanced FAS [36]. Blockade of STAT3 activation inhibits the glycolytic flux and decreases in PFKFB3 and HK2 expression in RA monocytes resulting in metabolic reprogramming and resolution of inflammation [118]. Furthermore, STAT3 differentially regulates RA FLS and human umbilical vein endothelial cells (HUVEC) metabolic and functional activity [130]. Finally, HIF1α and STAT3 interact with various key signalling molecules including NF-κB and Notch signalling through complex positive and negative feedback loops in the RA joint [10, 22, 25, 117, 131–134].

### AMPK and mTOR

One of the major cellular energy sensing pathways is the mechanistic target of rapamycin (mTOR) kinase, which is an evolutionary conserved serine-threonine kinase involved in controlling cellular growth, proliferation, and protein synthesis [135]. In environments of high glucose availability mTORC1 is phosphorylated and activates a number of downstream targets to promote anabolic processes, including protein, nucleotide, and lipid synthesis, while blocking the catabolic processes such as autophagy. However, in glucose-deprived environments, mTORC1 is inhibited, leading to a decrease in cellular growth and proliferation. The energy sensor AMP-activated protein kinase (AMPK) acts upstream of mTORC, regulating its activity in response to cellular glucose levels. Under condition of glucose starvation, AMPK inhibits mTORC1 through the phosphorylation and activation of the mTOR negative regulator tuberous sclerosis complex 2 (TSC2), and through the phosphorylation and inhibition of the mTORC1 component regulatory-associated protein of mTOR (Raptor) (Fig. 4). This response restores depleted cellular energy stores of ATP, leading to pathways that generate de novo ATP and inhibit energy-expensive pathways of ATP utilization, including inflammatory pathways. In the context of RA, studies have shown that B cell induction of inflammatory Th subsets and glucose utilization is mediated by mTOR activation [136]. mTOR activation in RA B cells induces IL-6 and RANKL expression, key mediators involved in the inflammatory and bone destructive response in RA [137], with this study also demonstrating that mTOR phosphorylation in CXCR3+ memory B cells correlates with RA disease activity [137]. Combination of rapamycin–metformin significantly attenuates inflammatory responses in RA via regulation of the Th17/Treg ratio and subsequent suppression of proinflammatory cytokines [138]. Consistent with this, the mTOR inhibitor Sirolimus promotes expansion of Treg cells in RA patients, restoring the Th17/Treg balance in favour of resolution of inflammation [139]. IL-9-induced proliferation of T cells is dependent on PI3K/Akt/mTOR signalling pathways [140]. Furthermore, RA T cells display a defect in N-myristoyltransferase (NMT) function, the effects of which inhibit AMPK activation, allowing activation of mTORC1 signalling and subsequent pro-inflammatory Th1 and Th17 differentiation. NMT1 overexpression induces AMPK activation leading to suppression of synovial inflammation [141].

Numerous studies have also suggested a role for mTOR signalling which mediates mechanisms involved in driving erosive disease in RA, through regulation of RA FLS invasion and proliferation [142, 143]. This is supported by studies demonstrating rapamycin and/or curcumin inhibits inflammation and synovial hyperplasia by reducing the number of invading RA FLS via the mTOR pathway [143, 144]. Furthermore, IL-1β induces the amino acid transporter SLC7A5 in RA FLS, which in turn promotes MMP3 and MMP13 secretion through NF-κB and mTOR-P70S6K signalling activity [145]. Consistent with this, other studies have shown that RA FLS proliferation is mediated by IGF-IR/PI3K/AKT m-TOR signalling pathway [146], that knockdown of Raptor (a component of mTORC1) reduces IL-1β-induced MMP3 and MMP13 expression in human intervertebral discs [147], and that mTORC1 blockade reduces synovial osteoclast formation, thus protecting the joint from bone erosion [148]. Finally, TNF signalling has been shown to co-opt the mTOR pathway inducing IFNγ responses in RA FLS through differential NF-κB- and STAT-1 signalling [142], further emphasizing the complex signalling interactions involved in driving RA disease pathogenesis.

Metformin, the anti-diabetic drug, which indirectly activates AMPK, has been shown to mitigate disease in models of arthritis [149] via the inhibition of mTOR pathway and the suppression of NF-κB-mediated inflammatory cytokine production, paralleled by enhanced autophagic flux [150]. Metformin inhibits systemic inflammation and synovitis and plays a role in bone protection by inhibiting cartilage layer matrix degradation, osteoclast formation, and chondrocyte apoptosis [151]. Metformin decreases spontaneous secretion of pro-inflammatory mediators from *ex vivo* RA synovial explants [152]. Finally, in a double-blind placebo-controlled study, metformin potentiated the anti-rheumatic effect of methotrexate on disease activity in RA [153].

### Targeting metabolites

Therapeutic targeting of metabolites is a promising prospect for the treatment of rheumatic diseases (Table 1). However, many therapeutics already in use in RA and other inflammatory arthritides alter metabolites and metabolic signalling pathways both directly and indirectly. First-line treatments for RA such as glucocorticoids are anti-metabolic, affecting genes associated with glycolysis and mTOR pathways and regulate respiratory rate in RA peripheral blood mononuclear cells [160–163]. Indeed conventional synthetic disease-modifying anti-rheumatic drugs (DMARDs) [164], many biologic DMARDs and the anti-diabetic drug Metformin have been shown to modulate immune cell and stromal cell metabolism in different forms of inflammatory arthritis [10, 47, 81, 138, 149, 150, 152, 165–168]. Furthermore, several studies have shown that treatment with the AMPK inhibitor AICAR suppresses RA FLS invasive function [169], decreases spontaneous secretion of proinflammatory mediators IL-6, IL-8, and MCP-1 from *ex vivo* whole tissue synovial explant cultures, and inhibits the pro-invasive effect of CD4 T on the RA FLS, evident by reduced growth factor and MMPs expression, thus implicating AICAR as potential target for treatment of RA [155].

**Table 1:** Potential metabolic targets in RA

Target	Mechanism	References
Knockdown of the Lactate transporter Lactate dehydrogenase inhibitors	• Reduce RA FLS invasiveness • Inhibit T-cell effector function • Ameliorate Joint Inflammation in animal models of arthritis	• Fujii, *et al*. [29] • Kvacskay, *et al*. [30] • Haas, *et al*. [35] • Pucino, *et al*. [36] • Li, *et al*. [154]
Silencing Succinate receptor GPR91 Silencing Tubulin acetyltransferase	• Inhibit Macrophage activation • Inhibit DC activation and T-cell expansion • Inhibit T-cell effector function	• Littlewood-Evans, *et al*. [41] • Rubic, *et al*. [43]
Glutaminase 1 inhibition	• Th17 expansion • Reduces RA FLS invasiveness	• Xu, *et al*. [83] • Ueda, *et al*. [85] • Takahashi, *et al*. [80]
Anti-IDO	• Inhibits T-cell and B-cell responses • Ameliorates Joint Inflammation in animal models of arthritis	• Pigott, *et al*. [94] • Pigott, *et al*. [95] • Chen, *et al*. [97]
NAMPT inhibition (FK866)	• Ameliorates Joint Inflammation in animal models of arthritis	• Busso, *et al*. [107] • Evans, *et al*. [108]
AMPK inhibitors (mTOR Targeting)	• Inhibits RA FLS invasiveness • Inhibits T effector cell responses • Reduces the Th17/Treg ratio • Inhibits RA FLS invasiveness and osteoclast formation	• Chen, *et al*. [146] • Petrasca, *et al*. [155] • Wen, *et al*. [141] • Kim, *et al*. [138] • Cejka, *et al*. [148] • Son, *et al*. [149]
3-Bromopyruvate (3-BrPA) which targets H1/2	• Reduces RAFLS invasiveness • Inhibits T-cell effector cell function • Ameliorates Joint Inflammation in animal models of arthritis	• Szczuka, *et al*. [156] • Okano, *et al*. [157] • Bustamante, *et al*. [158]
L-kynurenine (IDO pathway)	• Ameliorates Joint Inflammation in animal models of arthritis	• Williams [159]

Studies have also shown that treatment with the lactate dehydrogenase A (LDHA) inhibitor GSK2837808A results in a shift in the dampened ATP/AMP ratio back to baseline and increased hyaluronic acid secretion in activated osteoarthritis fibroblasts [154], and can restore effector T-cell function even in the presence of lactate [37]. Targeting glutaminolysis via inhibition or genetic ablation of the enzyme glutaminase 1 (GLS1) inhibits RA FLS proliferation and ameliorates the severity of experimental autoimmune arthritis [80]. Succinate-CoA ligase-deficient T cells in RA display a hyperinflammatory phenotype and subsequent synovitis, an effect which is inhibited by targeted silencing of tubulin acetyltransferase [170]. 3-Bromopyruvate (3-BrPA) has also been implicated as a potential target for inflammatory arthritis due to its inhibitory effect on the glycolytic enzymes HK2, in addition to its effect on succinate dehydrogenase [156, 157]. Targeting either 3-BR-PA or FX11 (LDHA inhibitor) has also been shown to reduce lactate production in T-cell-stimulated FLS, paralleled by a decrease pro-inflammatory cytokine levels [30], in addition to reducing synovitis in animals of arthritis [158]. PFK15 an inhibitor of the key glycolytic enzyme PFKFB3 inhibits RA FLS function [171], in addition to synovitis in animal models of arthritis [33]. Targeted activation of LXR has been implicated in suppression of T-cell activation [172], with overexpression of PPARg also implicated in the suppression of synovial hyperplasia [60]. Finally, in animal models of arthritis, therapeutic administration of L-kynurenine (IDO pathway) significantly reduces clinical and synovial progression [159].

These studies highlight the potential of inhibiting metabolites and downstream metabolic pathways for therapeutic benefit in RA; however, there is a paucity in our knowledge due to a lack of data from the site of inflammation. Deeper analysis utilizing novel technologies applied to immune and stromal subsets residing within the synovial joint and in the systemic circulation at various stages of disease progression and response are crucial to determining the real therapeutic benefit of targeting metabolites and their downstream pathways in RA and other autoimmune diseases.

## Conclusions

The studies discussed in this review highlight the key contribution of metabolites in regulating immune and stromal cell function and subsequent impact on RA disease progression. Metabolites act as central hubs influencing multiple cellular functions and signalling pathways making them an attractive target for therapy. Several drugs already in use for the treatment of RA affect metabolites and metabolic signalling as discussed earlier. However, further studies are required to determine whether metabolomics could be used to identify predictive biomarkers for patients in terms of outcome and treatment response. Analysis of metabolites could enable patient stratification and differentiation between responders and non-responders. Metabolic communications between cell subsets in the synovial microenvironment are also poorly understood. The manner in which the various cellular subsets interact with each other and how they compete for or exchange metabolites in the synovium is yet to be uncovered in RA. Future metabolic studies should focus on the site of inflammation ‘the synovium’, incorporation of advanced technologies such as droplet-based single-cell RNA-seq for cellular profiling in tandem with single-cell metabolomics and imaging technologies which will facilitate unique insights and allow for the development of novel therapeutic molecules. Furthermore, analysis of metabolites in early disease such as in pre-RA (Arthralgia) patients will give an insight into the role they play in disease initiation and progression.
